# Environmental influences on the maximum quantum yield of terrestrial primary production

**DOI:** 10.1111/nph.71303

**Published:** 2026-05-27

**Authors:** David Sandoval, Victor Flo, Catherine Morfopoulos, Iain Colin Prentice

**Affiliations:** ^1^ Georgina Mace Centre for the Living Planet, Department of Life Sciences Imperial College London Ascot SL5 7PY UK; ^2^ Department of Animal Biology, Plant Biology and Ecology Autonomous University of Barcelona (UAB) Bellaterra (Cerdanyola del Vallès) Catalonia E08193 Spain; ^3^ CREAF Ecological and Forestry Applications Research Centre Bellaterra (Cerdanyola del Vallès) Catalonia E08193 Spain; ^4^ Department of Earth System Science Tsinghua University Beijing 100084 China

**Keywords:** aridity, b6f, efficiency, GPP, photosynthesis, quantum, temperature, yield

## Abstract

- Historically, terrestrial biosphere models (TBMs) have assigned the intrinsic (maximum) quantum yield of photosynthesis ($\phi_{0})$ a constant value for each plant functional type. However, experimental studies have shown that $\phi_{0}$ – when measured on light‐adapted leaves – depends on temperature. It is unclear whether this dependence is universal or biome‐specific; how it is manifested at the ecosystem level; and how it should be represented in TBMs.
- By fitting empirical light‐response curves to a global set of eddy‐covariance CO_2_ flux measurements and correcting for photorespiration, we inferred apparent, ecosystem‐level $\phi_{0}$ values and their temperature responses across a wide range of environments.
- The temperature response of the apparent ecosystem‐level $\phi_{0}$ follows a universal bell‐shaped curve. The shape of this curve does not markedly differ among biomes, but the maximum value of $\phi_{0}$ decreases with increasing aridity, its temperature optimum increases with increasing growth temperature, and its sensitivity to temperature increases as growth temperature declines.
- Our model for $\phi_{0}\left(T\right)$ aligns with recent theory highlighting the role of cytochrome *b*_6_ *f* in regulating the light reactions of photosynthesis. If implemented in TBMs, this model should allow better predictions of the responses of terrestrial ecosystem function to a warming climate.

## Graphical abstract

Temperature and aridity effects on terrestrial ecosystems' intrinsic quantum yield.

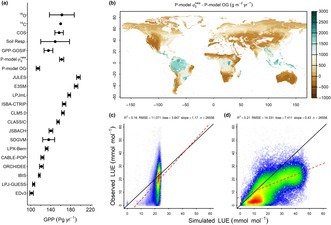

## Introduction

Uncertainties in the prediction of photosynthesis can cause large discrepancies in projections of ecosystem responses to global environmental change (Harrison *et al*., 2021). Most current terrestrial biosphere models (TBMs) (Fisher *et al*., 2014; Harrison *et al*., 2021) rely on the biochemical theory of photosynthesis developed by Farquhar, von Caemmerer and Berry (FvCB) (Farquhar *et al*., 1980). However, despite sharing the same core equations, ΤΒΜs have implemented them using varying conceptualisations of leaf‐to‐canopy scaling (from leaf‐level photosynthesis to ecosystem‐level gross primary production, GPP), and different assumptions about the values of key parameters that are not specified by the theory (Rogers *et al*., 2017). Hence, although TBMs based on FvCB might be expected to yield similar GPP estimates, in fact, they do not. Indeed, many models show systematic differences both from one another and from accepted observational ranges, especially regarding the response of GPP to temperature (Dietze, 2014; Prentice *et al*., 2015; Harrison *et al*., 2021).

One cause of variation among models' GPP estimates is the value or values assigned to a parameter of particular importance for the land carbon cycle (McNeall *et al*., 2024): the intrinsic quantum yield of photosynthetic carbon fixation ($\phi_{0}$). $\phi_{0}$ is the initial slope of the light‐response curve of photosynthesis under non‐photorespiratory conditions (high CO_2_ and low O_2_ partial pressure); thus, it sets the maximum efficiency of photosynthesis (Long *et al*., 1993; Rogers *et al*., 2017). We use the symbol $\phi_{0}$ here for the intrinsic, or maximum, quantum yield of CO_2_ assimilation at low light intensity to distinguish it from *φ* without a subscript, which denotes the quantum yield at low light intensity under ambient partial pressures of CO_2_ and O_2_. Both quantities are distinct from the maximum carboxylation rate (*V*_cmax_) and the maximum rate of electron transport (*J*_max_) as defined in the FvCB model, whose temperature dependencies have been studied far more extensively. Research on the light reactions of photosynthesis since the 1950s has established the theoretical maximum value for $\phi_{0}$ to be either 1/8 = 0.125, that is, one mole of assimilated CO_2_ requires at least eight moles of absorbed photons based on the NADPH requirement of carbon fixation (Warburg *et al*., 1950; Emerson, 1958; Walker, 1992; Hill & Govindjee., 2014), or 1/9 ≈ 0.111 based on the ATP requirement (Evans, 1987; Long *et al*., 1993). Almost all reported values have been obtained from measurements on leaves conducted in a non‐photorespiratory atmosphere, within a narrow temperature range (25–30°C) and with adequate water supply. Under these conditions, measured $\phi_{0}$ falls within the range of 0.07–0.125 mol CO_2_ mol^−1^ photon, with the highest values in shade‐adapted plants, and lower values in C_4_ compared to C_3_ plants (Ehleringer & Björkman, 1977; Ehleringer & Pearcy, 1983; Björkman & Demmig, 1987; Long *et al*., 1993; Singsaas *et al*., 2001; Skillman, 2008).

Despite limited knowledge of how $\phi_{0}$ responds to low or high temperatures and varying water availability, and how it upscales from leaves to ecosystems, it has generally been assumed that the available leaf‐level observations are representative and sufficient to infer globally applicable values for modelling purposes (Walker, 1992; Long *et al*., 1993; Singsaas *et al*., 2001). This assumption has led to $\phi_{0}$ being prescribed in TBMs as a constant value per plant functional type, with no variation with temperature (Rogers *et al*., 2017). Empirical light‐use efficiency (LUE) models that incorporate a downward regulation factor for ambient CO_2_ (e.g. Zhang *et al*., 2019; Bao *et al*., 2022; Cao *et al*., 2022) have similarly relied on prescribed, constant maximum LUE values for each plant functional type.

**Fig. 1:**
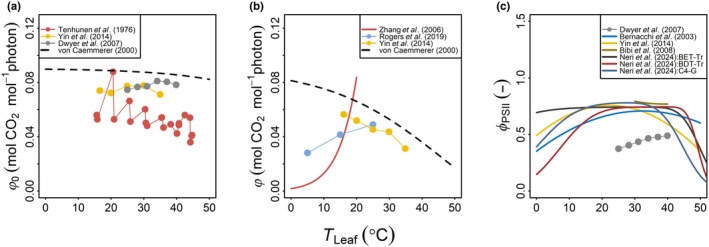
Maximum quantum yield responses to temperature from various sources. Connected dots represent observations, solid lines represent empirical models fitted to observations, and dashed lines represent predictions from the Farquhar, von Caemmerer and Berry (FvCB) theory (Eqn 1). (a) Non‐photorespiratory maximum quantum yield of CO_2_ assimilation (Tenhunen *et al*., 1976b; von Caemmerer, 2000; Dwyer *et al*., 2007; Yin *et al*., 2014); Eqn 1 forced here with CO_2_ set at 750 μmol mol^−1^ and 2% O_2_. (b) Ambient maximum quantum yield of CO_2_ assimilation (von Caemmerer, 2000; Zhang *et al*., 2006; Yin *et al*., 2014; Rogers *et al*., 2019); Eqn 1 forced here with CO_2_ set at 400 μmol mol^−1^ and 21% O_2_. (c) Quantum yield of Photosystem II in light‐adapted leaves from different studies (Bernacchi *et al*., 2003; Dwyer *et al*., 2007; Bibi *et al*., 2008; Yin *et al*., 2014; Neri *et al*., 2024). Selected PFTs from Neri *et al*. (2024): BET‐Tr, broadleaf evergreen tropical tree; BDT‐Tr, broadleaf deciduous tropical tree; C4‐G, C4 grass.

The temperature (in)dependence of $\phi_{0}$ can be analysed using a corollary to the FvCB model (Farquhar *et al*., 1980; von Caemmerer, 2000), which approximates the quantum yield of leaf‐level photosynthesis at low light as follows:

(Eqn 1) $$\phi \;\approx \;\lim_{I\to 0}\frac{\partial A}{\partial I}=\frac{c_{i}-\Gamma^{*}}{8c_{i}+16\Gamma^{*}}\left(1-f\right)\alpha =\frac{1}{8}m_{j}\left(1-f\right)\alpha$$

where A is the assimilation rate, I is the incident irradiance, $c_{i}$ is the intercellular CO_2_ partial pressure, α is the leaf absorptance, f is a correction factor for light quality (≈0.15; von Caemmerer, 2000) and $m_{j}=\left(c_{i}-\Gamma^{*}\right)/\left(c_{i}+2\Gamma^{*}\right)$ where $\Gamma^{*}$ is the photorespiratory compensation point, given by:

(Eqn 2) $$\Gamma^{*}=\frac{pO_{2}K_{c}V_{\text{omax}}}{2K_{o}V_{\text{cmax}}}$$

where *pO*_2_ is the partial pressure of oxygen, *V*_max_ and *K* are the maximum rates and Michaelis–Menten coefficients, respectively, and the subscripts _o_ and _c_ refer to oxygenation or carboxylation. Under nonphotorespiratory conditions $\Gamma^{*}$ becomes negligible; so $m_{j}$ approaches 1.0, and $\phi_{0}$ approaches $\frac{1}{8}\left(1-f\right)\alpha$ (Fig. 1a), independent of temperature. Under photorespiratory conditions ϕ is predicted only to decline with increasing temperature, due to the greater temperature sensitivity of *K*_o_ relative to *K*_c_.

However, these predictions are inconsistent with observations. Eqn 1 generally overestimates ϕ at low temperatures, under both non‐photorespiratory and ambient CO_2_ conditions (Fig. 1b); and as noted by Rogers *et al*. (2019) for Arctic plants, the observed change of ϕ with temperature below 25°C is opposite to that predicted, increasing rather than decreasing with temperature. This qualitative disagreement between theory and observations might explain some of the inconsistencies in the temperature responses of GPP predicted by FvCB‐based TBMs (Bloomfield *et al*., 2023).

In principle, $\phi_{0}$ reflects the emergent efficiency of the whole electron‐transport chain. According to a recent mechanistic description of the electron transport system (Johnson & Berry, 2021), this involves the efficiencies of both Photosystems I and II, with the cytochrome *b*_6_ *f* complex regulating the electron flow between the two in such a way as to minimise losses of absorbed light under low light while down‐regulating electron transport (to match the use of ATP and NADPH for carboxylation) under saturating light. The different elements of the coupled photosynthetic system work in tandem and have distinct temperature dependencies (Johnson *et al*., 2021; Johnson & Berry, 2021). In the FvCB theory, by contrast, the only temperature effect applied to the electron‐transport chain is to *J*_max_, which is empirically defined as the asymptotic rate of electron transport under saturating light and CO_2_ (Farquhar *et al*., 1980; Johnson *et al*., 2021) – implying that $\phi_{0}$ is not or barely temperature‐dependent, as suggested by Eqn 1.

Many empirical studies have focused on the initial stage of the electron‐transport chain, the large protein complex Photosystem II, whose quantum efficiency ($\Phi_{\text{PSII}}$), defined as the number of electrons transported per photon absorbed by PSII (as measured by pulse amplitude modulation fluorometry), is expected to be proportional to $\phi_{0}$ when the photosynthetic steady state is experimentally forced by actinic light under non‐photorespiratory conditions (Björkman & Demmig, 1987; Genty *et al*., 1989; Oberhuber *et al*., 1993). When measured on dark‐adapted leaves, $\Phi_{\text{PSII}}$ shows little or no temperature dependence. In light‐adapted leaves, however, $\Phi_{\text{PSII}}$ typically shows a distinct bell‐shaped response to temperature (Fig. 1c) in both C_3_ and C_4_ species (Dwyer *et al*., 2007). Fig. 1 also shows data (or empirical models fitted to data) on *φ*_0_ (Fig. 1a), derived from CO_2_ assimilation measured under non‐photorespiratory conditions, and *φ* (Fig. 1b), from CO_2_ assimilation under ambient conditions, indicating a range of responses to temperature but broadly suggestive of a unimodal pattern similar to that shown for $\Phi_{\text{PSII}}$ in Fig. 1(c).

The large differences among the various published apparent quantum yield responses of *φ*_0_ and *φ* to temperature, the limited temperature range of many data sets at the leaf scale, and the disagreement between standard theory predictions and observations suggest that a more comprehensive global analysis would be helpful for the representation of *φ*_0_ in TBMs. Here we derive apparent, ecosystem‐level values of ϕ, and thence $\phi_{0}$, from the slopes at low light of the light‐response curves of net ecosystem exchange (NEE) as observed across the global network of eddy‐covariance flux towers. We fit a universal empirical model to this response, and show how the model's parameters are influenced by adaptation to different growth environments.

## Materials and Methods

We exploited the abundance of half‐hourly NEE data available from eddy‐covariance CO_2_ flux measurements, together with *in situ* observations of the fraction of absorbed photosynthetic active radiation (fAPAR) made at 53 sites in the Ameriflux network (Novick *et al*., 2018). We estimated fAPAR from measured above‐ and below‐canopy Photosynthetic Photon Flux Density (PPFD, μmol m^−2^ s^−1^) as follows (Carrara *et al*., 2018):

(Eqn 3) $$\text{fAPAR}=\frac{{\text{PPFD}}_{\text{IN}}-{\text{PPFD}}_{\text{OUT}}-{{\text{PPFD}}_{\mathrm{BC}}}_{\text{IN}}\left(1-\alpha_{\text{soil}}\right)}{{\text{PPFD}}_{\text{IN}}}$$

where PPFD_IN_ is the incoming (incident) PPFD and PPFD_OUT_ is the outgoing PPFD at the top of the canopy, and ${{\text{PPFD}}_{\text{BC}}}_{\text{IN}}$ is the incoming PPFD below the canopy. $\alpha_{\text{soil}}$ is the soil albedo in the visible light spectrum, taken here as 0.17 (Feister & Grewe, 1995). Absorbed PPFD (*I*_abs_) was estimated as the product of PPFD_IN_ and fAPAR. We additionally used a larger, globally distributed set of 310 sites (Pastorello *et al*., 2020; Hufkens, 2022; Ukkola *et al*., 2022) using remotely sensed fAPAR (Myneni *et al*., 2015) from the MODIS MCD15A3 product (Myneni *et al*., 2015), interpolated to daily timesteps.

**Fig. 2:**
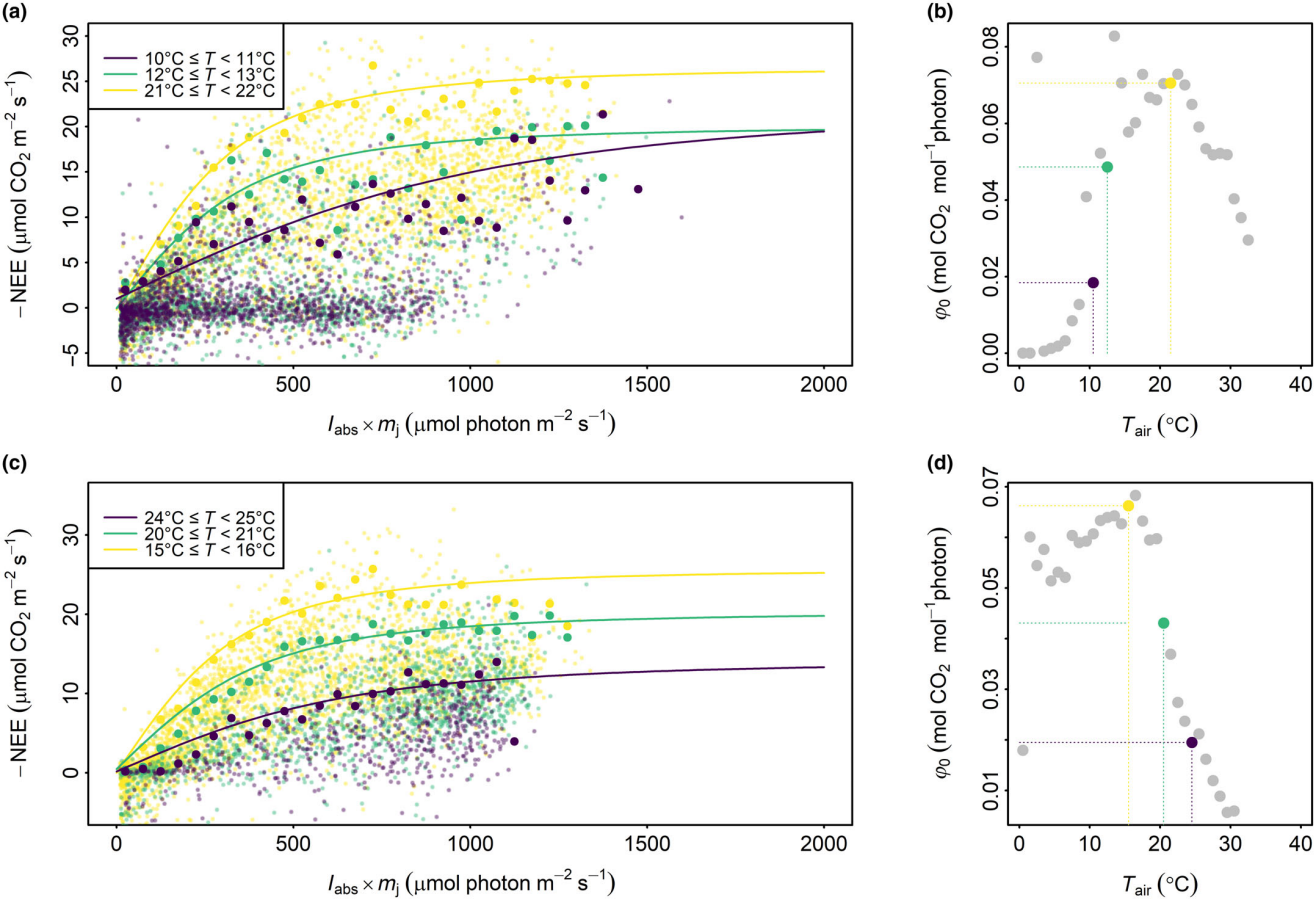
Method used to extract $\phi_{0}\left(T\right)$. Three 1.0°C intervals with different colours are shown as an example for the Harvard Forest site US‐xHa (Biome: deciduous broadleaf forest, mean annual temperature = 6.6°C, mean annual precipitation = 1071 mm) (a,b) and British Columbia, Douglas‐fir stand site CA‐Ca1 (Biome: evergreen needleleaf forest, mean annual temperature = 9.9°C, mean annual precipitation = 1369 mm) (c, d). (a, c) Point clouds per temperature bin: larger points denote the 95^th^ percentile, and solid lines are Eqn 4 fitted to these 95^th^ percentiles. (b, d) $\phi_{0}$ extracted from the fitted Eqn 4, as shown in (a, c). NEE, net ecosystem exchange.

For each site, we estimated $\phi_{0}$ values within 1°C temperature bins using Nonlinear Least Squares. To capture the maximum efficiency and avoid limitations caused by factors other than temperature and light (e.g. soil moisture), we used only the upper envelope (95^th^ percentile) of the data; and only data points flagged as daytime good‐quality observations were used. To account for the curvature of the light‐response curve and ecosystem respiration (Re), we fitted the following hyperbolic equation to the NEE data (Smith, 1937; Stocker *et al*., 2020):

(Eqn 4) −NEE=A−Re=ϕ0Iabsmj1+ϕ0IabsAsat2−Re

where *A*_sat_ is the asymptotic value of GPP under saturating light. The factor *m*_j_ corrects for photorespiratory conditions and the effect of vapour pressure deficit $\left(D\right)$ on stomatal opening, following the least‐cost hypothesis:

(Eqn 5) $$m_{i}=\frac{c_{i}-\Gamma^{*}}{c_{i}+2\Gamma^{*}}=\frac{c_{a}-\Gamma^{*}}{c_{a}+2\Gamma^{*}+3\Gamma^{*}\sqrt{\frac{1.6\eta^{*}D}{\beta \left(K+\Gamma^{*}\right)}}}$$

where *c*_a_ is the ambient partial pressure of CO_2_; *K* = *K*_c_ (1 + *p*O_2_/*K*_o_) is the effective Michaelis constant of Rubisco; η* is the viscosity of water, normalised by its value at 25°C; *D* is the vapour pressure deficit; and *β* is a constant, estimated as 146 (Stocker *et al*., 2020) based on global leaf δ^13^C data. The method is illustrated in Fig. 2.

Once $\phi_{0}$ was found at each temperature bin, we fitted $\phi_{0}=f\left(T\right)$ to a peaked Arrhenius equation (Eqns 6, 7; Kattge & Knorr, 2007) by using the classic Nelder–Mead optimisation algorithm (Nelder & Mead, 1965) with the Nash–Sutcliffe (NSE) coefficient (Nash & Sutcliffe, 1970), which relates the variance of the residuals to the variance of the data as the objective function, then we repeated this procedure for all sites:

(Eqn 6) $$T_{\mathrm{opt}}=\frac{H_{d}}{\Delta S-\text{Rlog}\left(\frac{H_{a}}{H_{d}-H_{a}}\right)}$$

(Eqn 7) $$\phi_{0}\left(T\right)=\hat{\phi_{0}}\frac{H_{d}\;\exp\left(\frac{H_{a}\left(T_{\mathrm{air}}-T_{\mathrm{opt}}\right)}{T_{\mathrm{air}}\;R\;T_{\mathrm{opt}}}\right)}{H_{d}-H_{a}\left(1-\exp\left(\frac{H_{d}\;\left(T_{\mathrm{air}}-T_{\mathrm{opt}}\right)}{T_{\mathrm{air}}\;R\;T_{\mathrm{opt}}}\right)\right)}$$

where $T_{\mathrm{opt}}$ (K) is the temperature at the peak, $H_{a}$ (J mol^−1^) is the activation energy, $H_{d}$ (J mol^−1^) is the deactivation energy, ΔS (J mol^−1^ K^−1^) is the entropy change between the ground and transition states of the reaction, $\hat{\phi_{0}}$ is the intrinsic quantum yield at $T_{\mathrm{opt}}$, and R (J mol^−1^ K^−1^) is the universal gas constant.

To test whether the site's bioclimate shapes this response to temperature, we fitted nonlinear models using bioclimatic indices explaining ΔS, $T_{\mathrm{opt}}$ and $\hat{\phi_{0}}$ parameters from the peaked Arrhenius equation with 10 000 bootstrap resampling iterations. The bioclimatic metrics tested were the aridity index $\left(\mathrm{AI}=\mathrm{PET}/P\right)$, evaporative index $\left(\mathrm{EI}=\mathrm{ET}/P\right)$, evaporative fraction $\left(\mathrm{EF}=\mathrm{ET}/\mathrm{PET}\right)$ and mean temperature during growth days (mGDD_0_), where PET is the mean annual potential evapotranspiration, P is the mean annual precipitation, and ET is the mean annual actual evapotranspiration. PET was estimated using the SPLASH v2.0 model (Sandoval *et al*., 2024), which accounts for surface temperature feedback on the net radiation. Actual evapotranspiration was estimated by converting the latent heat observed at the tower using the latent heat of vaporisation with corrections for local temperature and pressure. Growth days are defined as days with mean temperatures > 0°C. Mean growth temperature (mGDD_0_) was estimated as the average temperature during those days. All the environmental data used to estimate the bioclimatic metrics were measured *in situ*, except for sites where the records were shorter than 5 yr. In such cases, data from ERA5 (Muñoz‐Sabater, 2019) were used instead.

To compare our model, theoretical simulations of $\phi_{0}$ were carried out using the model and code described in Johnson *et al*. (2021) with default parameters for *Populus fremontii* as used in Johnson & Berry (2021). Bioclimatic metrics for the sites described in Johnson & Berry (2021) and Rogers *et al*. (2019) were calculated using data from ERA5 (Muñoz‐Sabater, 2019) and Splash v.2.0 (Sandoval *et al*., 2024). To evaluate the effect of the new formulation on global GPP $\phi_{0}\left(T\right)$ was implemented in the P model and global simulations were run with both the original which applies Bernacchi *et al*. (2003) $\phi_{0}\left(T\right)$ correction, and the new formulation.

## Results

**Fig. 3:**
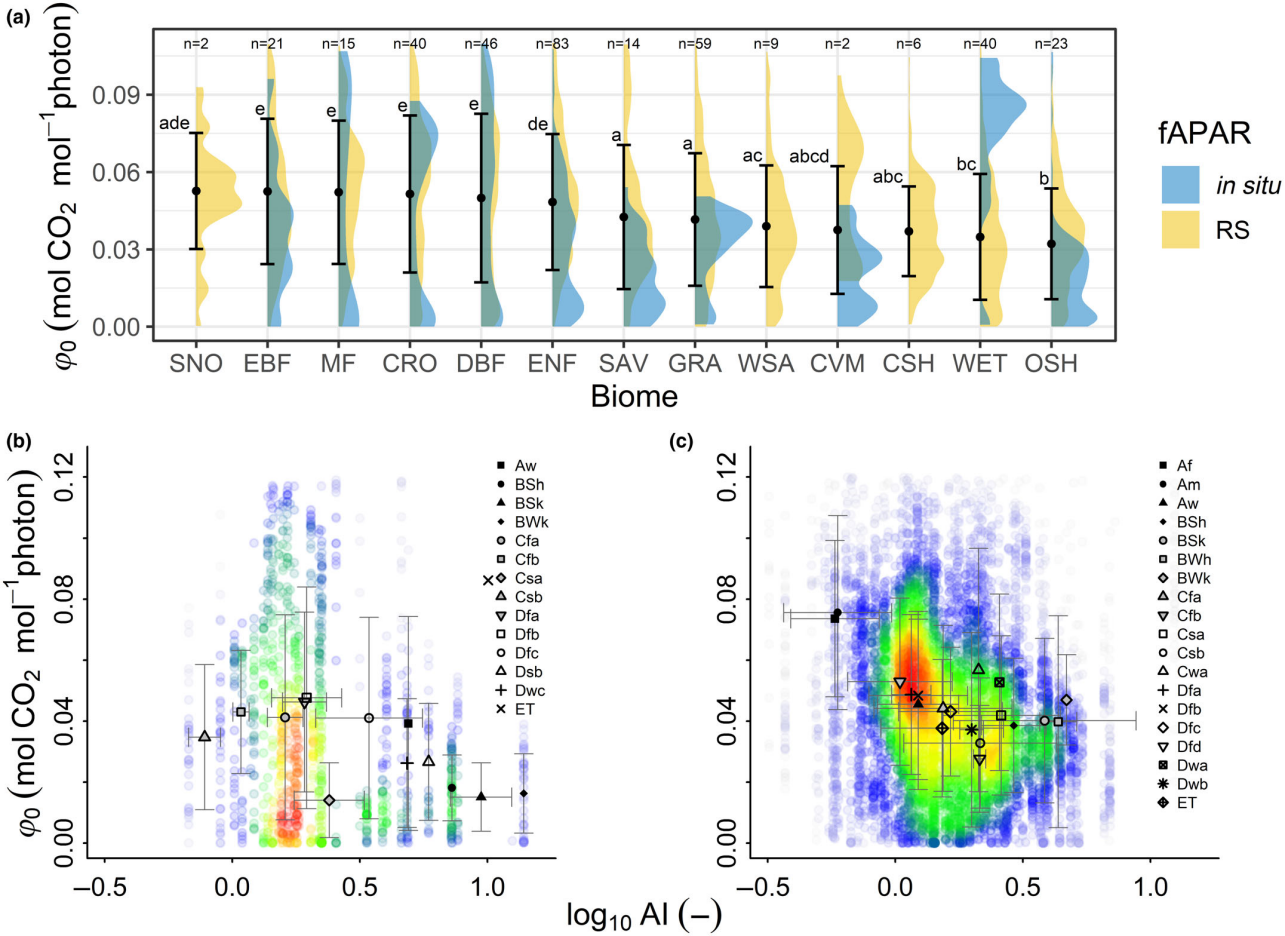
Observed $\phi_{0}$ inferred by using *in situ* and remotely sensed fAPAR. (a) Probability distribution of $\phi_{0}$ per biome and fraction of absorbed photosynthetic active radiation (fAPAR) source. The symbols and the black bars denote the mean and SD per biome, based on combined fAPAR sources. *Post hoc* Tukey test results at the top of the bars and the number of sites at the top of the distributions. (b) $\phi_{0}$ as a function of aridity index from *in situ* fAPAR sites. (c) $\phi_{0}$ as a function of aridity index from RS fAPAR sites. Symbols in (b, c) show the average $\phi_{0}$ values per climate zone and the colours denote point density. Bars denote standard deviation per biome.

$\phi_{0}$ varied considerably across biomes and climate zones. Mean values showed a smooth transition between biomes, with higher values in forests and lower values in grasslands and shrublands (Fig. 3a). Examining this transition using climate zones, a marked pattern emerged, driven by the aridity gradient. Tropical rainforest (Af) and tropical monsoon (Am) climates appeared at the upper end of the $\phi_{0}$ axis, followed by sites with ‘no dry season’ (*f* types), and then by sites with dry/wet seasonality (*w* and *s* types). Finally, sites from arid climates (BW*) and steppe appeared in the lower end (BS*) (Fig. 3b,c).

**Fig. 4:**
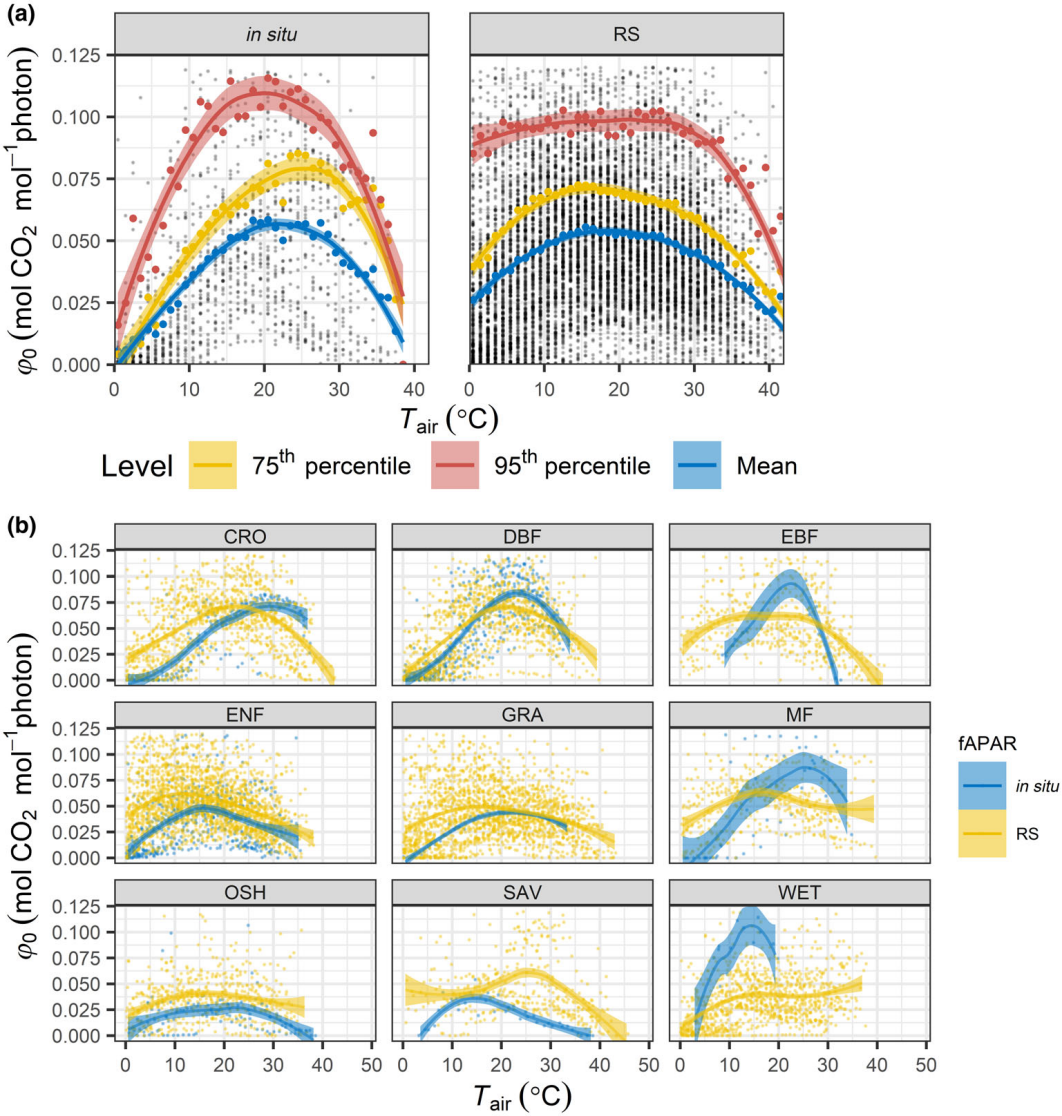
$\phi_{0}$ responses to temperature using all data pooled. The ribbons show a LOESS smoothing function including its 95% confidence intervals. (a) Pooled observations from sites with *in situ* fraction of absorbed photosynthetic active radiation (fAPAR) and sites with remotely sensed fAPAR. (b) Pooled observations per biome and fAPAR source.

The response of $\phi_{0}$ to temperature showed a bell‐shaped pattern across all sites (Fig. 4a). In general, the average $\phi_{0}$ steadily increased up to a maximum, followed by a sharper drop. This pattern also emerged using the 75^th^ and 95^th^ percentiles of $\phi_{0}$ (Fig. 4a). The global maximum efficiency, defined based on the 95^th^ percentile, was 0.111 ± 0.003 mol CO_2_ mol^−1^ photon and was located at the optimal temperature of 20.46 ± 0.37°C. The bell‐shaped response remained consistent across biomes with the *in situ* fAPAR data, while with the RS fAPAR wetlands and savannas showed inconsistencies (Fig. 4b). Open shrublands (OSH) showed lower $\phi_{0}$ values and lower sensitivity to *T*_air_, whereas mixed forests (MF) showed the highest $\phi_{0}$ values and higher sensitivity to *T*_air_.

These patterns are less pronounced when the (temporally sparse) remote‐sensing fAPAR data are used. In ENF, GRA and evergreen broadleaf forests (EBF) biomes, remotely sensed fAPAR produces higher $\phi_{0}$ at low temperatures – possibly indicating multiple optimal temperatures within each biome. In biomes where about the same temperature range was covered by both datasets (DBF, MF and OSH), the temperature sensitivity of $\phi_{0}\left(T\right)$ was also similar between the datasets.

**Fig. 5:**
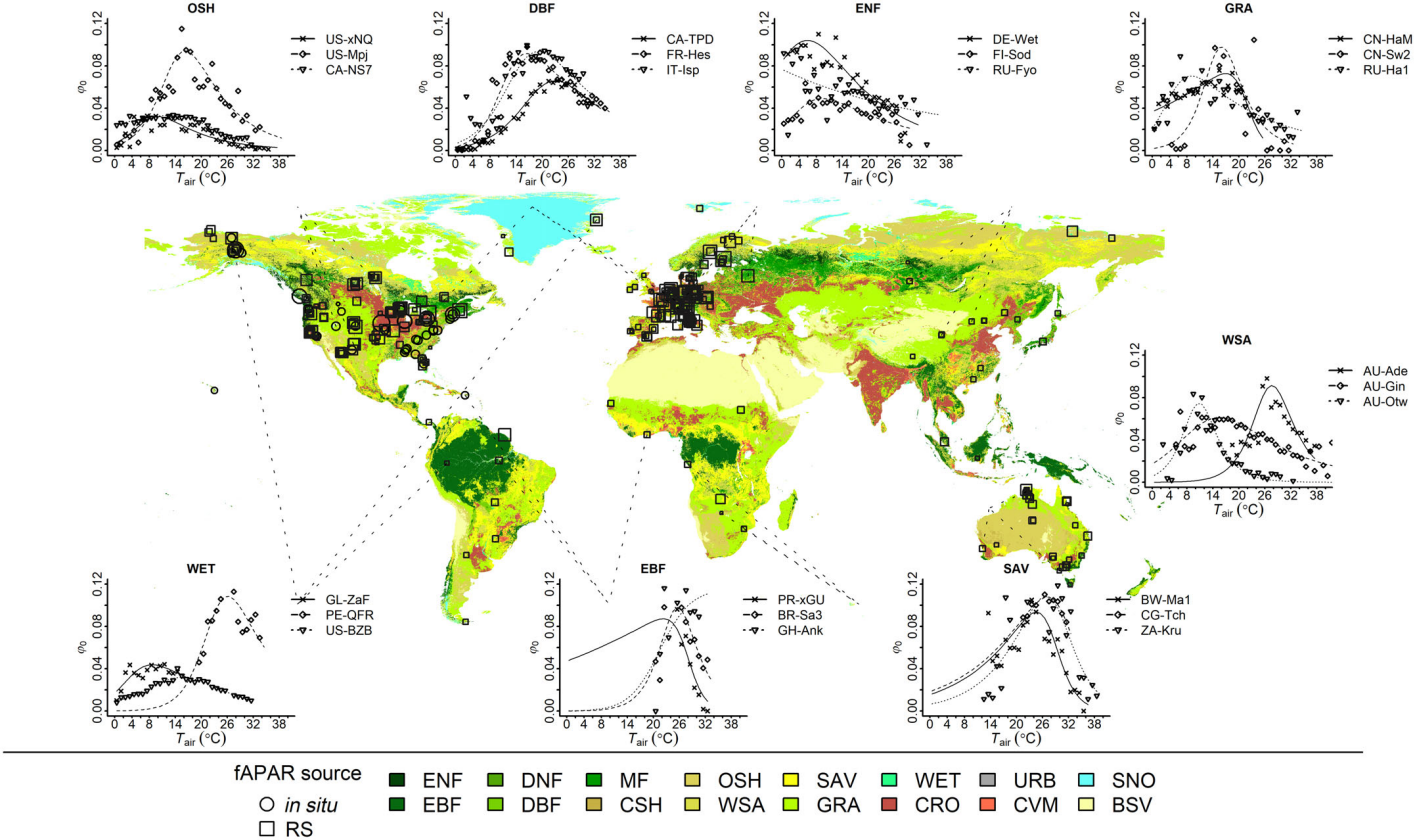
$\phi_{0}$ responses to temperature using peaked Arrhenius curves. Observation (points) and predictions (lines) from selected sites.

We further analysed the temperature response at individual sites using the fitted peaked Arrhenius curves. The shapes of the response were consistent regardless of the location, biome, or fAPAR source. However, different temperature sensitivities and optimal temperatures emerged. For example, wetlands (WET) as depicted in Fig. 5 show the extreme difference between a wetland in the Peruvian rain forest (PE‐QFR) and a wetland in Greenland (GL‐Zaf).

**Fig. 6:**
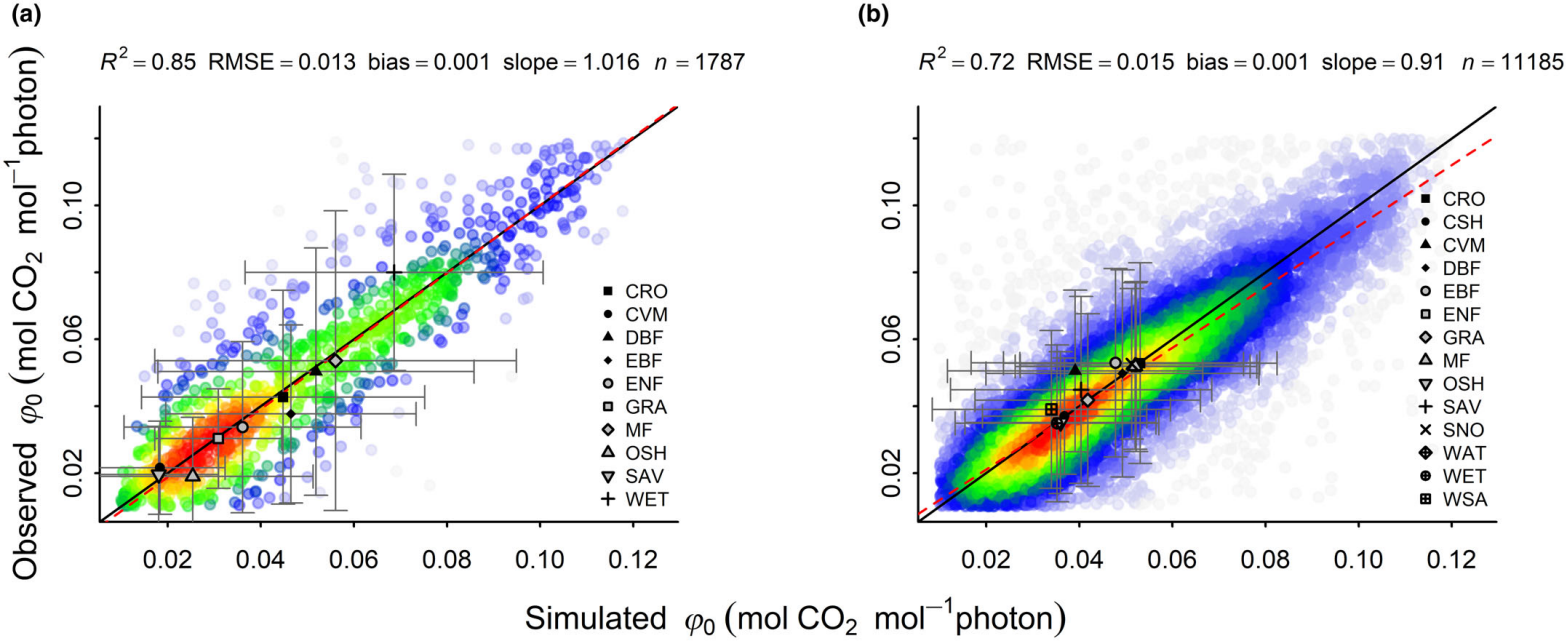
Correlations of observed and simulated $\phi_{0}\left(T\right)$ using peaked Arrhenius curves. (a) using *in situ* fraction of absorbed photosynthetic active radiation (fAPAR) observations. (b) using remotely sensed fAPAR observations. Colours denote point density. Black symbols and bars denote the mean and SD per biome.

Globally, the curves fitted site by site to the peaked Arrhenius equation were able to explain $\phi_{0}\left(T\right)$ with reasonably good accuracy. Performance was slightly better for the *in situ* fAPAR, explaining 85% of the variation while explaining 72% of the variation for the *RS* fAPAR. Most of the mean values (observed and simulated) for biomes fell close to the 1 : 1 line, except for OSH, which was slightly overestimated and WET was underestimated (Fig. 6a).

**Fig. 7:**
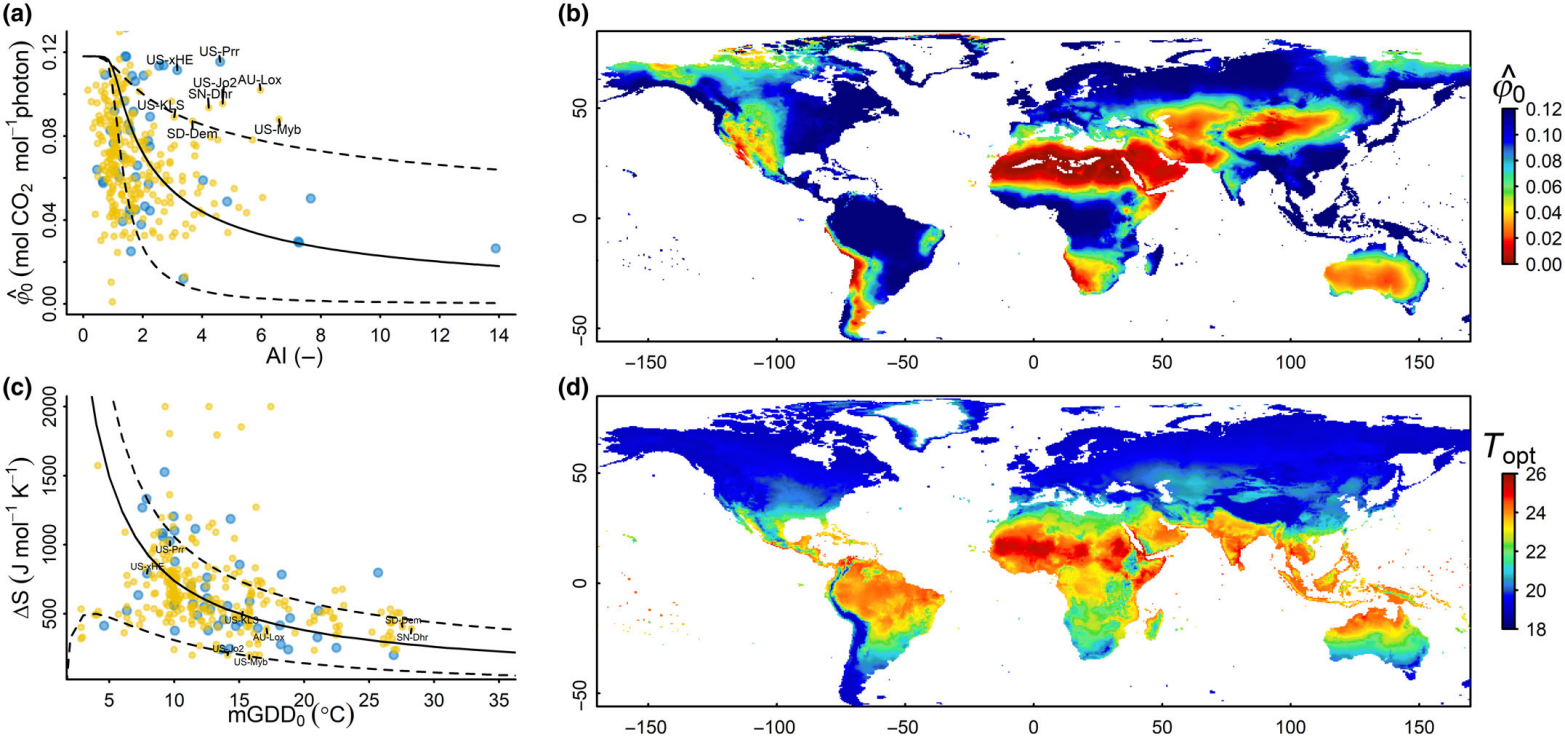
Bioclimatic patterns of the peaked Arrhenius parameters. The colours in the scatter plots refer to fraction of absorbed photosynthetic active radiation (fAPAR) measured *in situ* or from remote sensing. (a) `*ϕ*_0_ response to aridity: solid line regression, dashed lines 95% confidence intervals. (b) Global prediction of `*ϕ*_0_ using Eqn 8. (c) Entropy change (Δ*S*) variation with growth temperature: solid line regression, dashed lines 95% confidence intervals. (d) Global prediction of optimum temperature for $\phi_{0}$ using Eqn 9.

The peak $\phi_{0}\;\left(\hat{\phi_{0}}\right)$ showed considerable variation among sites, while $H_{a}$ was relatively stable among sites. For most (350 sites) $H_{a}$ was *c*. 70.9 kJ mol^−1^. $H_{d}$ varied from *c*. 111 kJ mol^−1^ to 351 kJ mol^−1^, while Δ*S* varied from *c*. 0.2 to >1.5 kJ mol^−1^ K^−1^ and was highly correlated to $H_{d}$. $\hat{\phi_{0}}$ decreased from a plateau when AI ≤ 1 with increasing aridity following an inverse sigmoid response (Eqn 8):

(Eqn 8) $$\hat{\phi_{0}}=\frac{0.111}{{\left(1+{\mathrm{AI}}^{m}\right)}^{n}}$$

where *m* = 4.09 (95% CI: 4.07–9.99) and *n* = 0.12 (95% CI: 0.050–0.169). Thus $\hat{\phi_{0}}$ shows a steep decay from 0.111 when AI > 1, stabilising (when AI > 8) at a value *c*. 0.03 (Fig. 7a). Some of the sites that fall outside the 95% confidence intervals are tagged in the plots and discussed in the next section. The global average of $\hat{\phi_{0}}$, according to the prediction from Eqn 8 (Fig. 7b), was 0.08 mol CO_2_ mol^−1^ photon.

ΔS decreases non‐linearly, approaching stability for mGDD_0_ > 30°C, as described by Eqn 9, where $\Delta S_{0}$ = 3468.2 (95% CI: 1523.9–6408.2) and kS = 0.668 (95% CI: 0.321–0.639):

(Eqn 9) $$\Delta S=\Delta S_{0}{\text{mGDD}}_{0}^{‐\mathrm{kS}}$$

Thus, replacing Eqn (9) in Eqn (6) produces $T_{\mathrm{opt}}$ within a range from *c*. 17°C in boreal regions to *c*. 26°C in the tropics (Fig. 7d), which also results in a rate of change $\frac{\Delta T_{\mathrm{opt}}}{\text{ΔmGD}D_{0}}=0.35$.

## Discussion

We derived apparent $\phi_{0}$ at the ecosystem level for globally distributed sites, using half‐hourly *in situ* measurements of fAPAR and eddy‐covariance measurements of CO_2_ exchange. We found that it shows a bell‐shaped response with temperature, and this pattern remains consistent across all individual sites analysed, even when remotely sensed fAPAR was used in the absence of *in situ* fAPAR measurements (Figs 4a, 5). However, this pattern was less consistent when the data were grouped per biome (Fig. 4b), suggesting that the temperature response curve for $\phi_{0}$ is not biome‐specific but instead gradually modulated by bioclimate. In particular, increasing aridity reduces $\hat{\phi_{0}}$, while higher growth temperatures shift the optimum temperature upward and simultaneously decrease the sensitivity of $\phi_{0}$ to instantaneous temperature. The use of absorbed rather than incident radiation should implicitly account for the effect of high diffuse fractions during low sun angles and their confounding effect with low temperatures. This effect should be clearer with the *in situ* fAPAR observations, which are higher under greater diffuse light, independently of temperature. Since MODIS overpasses occur at 10:30 h and 13:30 h, it is unlikely that they captured early‐morning periods of high diffuse light and their effect on fAPAR. However, this remains a caveat, as no data were available on the partitioning between diffuse and direct radiation.

No previous studies have reported $\phi_{0}$ at ecosystem scale. However, in our analysis, the fitted maximum value of $\phi_{0}$ (0.111 mol CO_2_ mol^−1^ photon) is identical to the theoretical maximum limited by ATP generation of 0.111 (Long *et al*., 1993) and to the maximum observed by Walker (1992). It also closely matches other values reported for single leaves under unstressed conditions including Björkman & Demmig (1987) (0.106 on average for C3 plants and 0.112 for conifers), Skillman (2008) (0.106) and Singsaas *et al*. (2001) (0.108).

Our analyses show that $\phi_{0}$ is generally higher in forests, followed by grasslands and shrublands, suggesting that $\phi_{0}$ varies among species from different life forms – in line with the observations by Singsaas *et al*. (2001), who noted that even a 100% variation in photorespiration plus alternative electron sinks could not fully explain the observed variation in $\phi_{0}$ among species. This result contrasts with the findings of Björkman & Demmig (1987), Walker (1992) and Long *et al*. (1993), who suggest an almost constant $\phi_{0}$ among all species, regardless of their life form.

*φ*₀ is, by definition, a conservative quantity that does not scale with the size of the system; therefore, *φ*₀__leaf_ ≈ *φ*₀__canopy_ (Supporting Information Notes S1). In theory, in a closed canopy, leaves at the top receive more light than those at the bottom, creating a vertical gradient in light exposure and assimilation. Thus, if measurements are taken simultaneously along a vertical profile, pairs of absorbed light–assimilation values should fall along the same light‐response curve, all else being equal (Fig. S1). However, many physiological variations occur within canopy vertical profiles that we do not account for, which represents a caveat of our analysis. Nonetheless, we expect that our estimate of ecosystem *φ*₀ provides a reasonable approximation of the average leaf‐level *φ*₀ for use in global modelling.

Another source of variation could be attributed to different proportions of C3/C4 plants which have distinct $\phi_{0}$ (Oberhuber *et al*., 1993), with higher proportions of C4, Eqn (5) might overestimate $\phi_{0}$ in tropical grasslands and savannas due to C4 plants' low photorespiration.

**Fig. 8:**
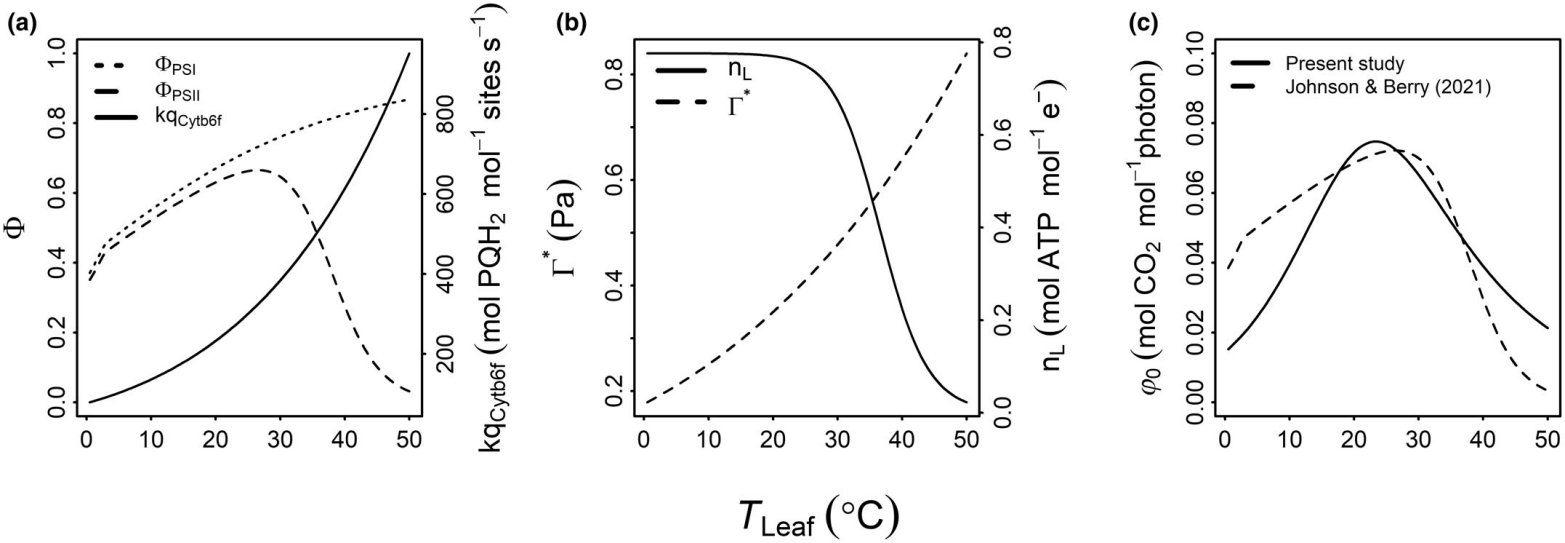
Theoretical responses of selected electron‐transport chain components to temperature, following Johnson & Berry (2021). Simulations were carried out under non‐photorespiratory conditions: CO_2_ = 700 μmol mol^−1^, *pO*_*2*_ = 2029 Pa, and low light (150 $\text{μmol}\;m^{-2}\;s^{-1}$). (a) Efficiencies of Photosystems I and II and the catalytic constant for cytochrome *b*_6_ *f*. (b) The photorespiratory compensation point and the coupling efficiency of linear electron flow to the proton circuit (ηL). (c) $\phi_{0}\left(T\right)$ predictions from theory and from the present study.

Our findings of a universal peaked response to temperature are consistent with the predictions from the theory developed by Johnson & Berry (2021) (Fig. 8c) – supporting the idea that the bell‐shaped response of $\phi_{0}$ to temperature is a general plant response which propagates to the ecosystem, thus creating a general ecosystem response to temperature.

In the Johnson‐Berry theory, cytochrome *b*_6_ *f* (Cyt *b*_6_ *f*) modulates the transfer of electrons from plastoquinol (PQH2) to oxidised plastocyanin (PCox) at a rate *kq* (Fig. 8a), redistributing excitation energy between photosystems and diverting energy to thermal dissipation. Cyt *b*_6_ *f* also couples this electron transfer to the proton circuit from the stroma into the lumen, modulating the coupling between linear and cyclic electron flux (Johnson & Berry, 2021). When light is limiting, Cyt *b*_6_ *f* activity is at its maximum and so *kq* depends primarily on temperature, following a regular Arrhenius response. Most of the electron flow passing through Cyt *b*_6_ *f*, at limiting light, and connecting to the proton circuit, is thought to be linear; this efficiency decays exponentially at high temperatures (Fig. 8b; Johnson & Berry, 2021).

Low $\phi_{0}$ at low temperature could be explained by the accumulation of sustained nonphotochemical quenching (NPQ) (Porcar‐Castell, 2011) or by the rate of repair of Photosystem II, which slows down at low temperature (Greer *et al*., 1986; Mattila *et al*., 2020). If this rate of repair becomes slower than the rate of PSII damage, it could lead to photoinhibition even under low light (Osmond, 1994; Tyystjärvi & Aro, 1996). This damage‐repair imbalance is also documented when the rate of damage is enhanced by high temperature (Murata *et al*., 2007; Sage & Kubien, 2007). It has been reported that PSI activity decreases at low temperatures only in cold‐sensitive plants (Sonoike, 1999), otherwise it is thermally stable. Its inactivation does not occur before the complete inactivation of PSII (Berry & Bjorkman, 1980; Oberhuber & Edwards, 1993).

Although we corrected for stomatal closure and photorespiration (Eqn 5), implicitly accounting for Rubisco deactivation at high temperature, other factors could affect $\phi_{0}\left(T\right)$ such as the solubility of CO_2_ in water: this decreases as temperature increases, reducing the CO_2_ concentration in C_3_ plants and favouring oxygenation by Rubisco, as noted by Ehleringer & Björkman (1977). However, this effect is counterbalanced by the increase in CO_2_ diffusivity and hence the increase of mesophyll conductance (von Caemmerer & Evans, 2015).

According to Eqn 6, respiration offsets the light‐response curve in each temperature bin without affecting the slope $\phi_{0}$. Nonetheless, we double‐checked our results by repeating the analysis using GPP from the FLUXNET dataset and setting respiration to zero, producing very similar results (Fig. S2).

Our results indicate that the maximum quantum efficiency $\hat{\phi_{0}}$ at the ecosystem level decreases as climatological aridity increases, consistent with the observations of Fu *et al*. (2022) regarding the maximum evaporative fraction, and with Mengoli *et al*. (2023) regarding the maximum LUE of GPP. Several explanations are possible, including: (a) persistent effects of drought on the photosynthetic apparatus, such as damage to the light‐harvesting complexes of PSII and reduction in the size of the PSI antenna (Hu *et al*., 2023); (b) high leaf mass per area (Wright *et al*., 2004), typical in plants adapted to aridity, favouring photoprotective thermal dissipation over photochemistry (Adams *et al*., 1987; Valladares *et al*., 2000); (c) coordination of hydraulic and allocation traits (Flo *et al*., 2021) such that plants of dry environments minimise hydraulic risk (Bassiouni *et al*., 2023). Variation of $\hat{\phi_{0}}$ with other bioclimatic variables was not significant (Figs S3, S4).

A few sites, however, showed relatively high $\hat{\phi_{0}}$ at AI > 1. Although we cannot account for every case, there are many possible reasons why local conditions might differ from the bioclimate of the surrounding regions. For example, some sites such as AU‐Lox (Stevens *et al*., 2011) and US‐KLS (Ji *et al*., 2021) are irrigated; while others, such as SN‐Dhr (Tagesson *et al*., 2015) and US‐Jo2 (Pérez‐Ruiz *et al*., 2022), experience a strong seasonal concentration of precipitation. Some sites have access to groundwater, like SD‐Dem (Ardo *et al*., 2008), or are man‐made wetlands, like US‐Myb (Eichelmann *et al*., 2018). Other sites show a high PET/P ratio, but a significant portion of the energy is used for melting snow and thawing permafrost such as US‐xHE (Osterkamp *et al*., 2009) and US‐Prr (Nakai *et al*., 2013).

Activation energies for *φ*_0_ showed little variation among sites; this is also the case for the activation energies of *V*_cmax_ and *J*_max_, as reported by Kattge & Knorr (2007). Δ*S* decreases with increasing growth temperature, as also reported for *V*_cmax_ and *J*_max_ by Kattge & Knorr (2007) and Kumarathunge *et al*. (2019). Variation in ΔS shifts the optimum temperature by 0.35°C per degree of growth temperature and simultaneously reduces the sensitivity of $\phi_{0}$ to T, as also noted by Yin & Struik (2009). This response is consistent with the observations of increased thermal stability of PSII at higher growth temperatures due to changes in thylakoid membrane lipids (Berry & Bjorkman, 1980). It is also consistent with the effect of higher growth temperatures on the accumulation of zeaxanthin, which enhances nonphotochemical quenching and thermal energy dissipation in PSII (Demmig‐Adams *et al*., 1989; Busch *et al*., 2007). Overall, the variations of ΔS and $\hat{\phi_{0}}$ with mGDD_0_ and aridity, respectively, support our general hypothesis that the temperature response of $\phi_{0}\left(T\right)$ depends on adaptation to bioclimate.

We replicated our analysis on a year‐by‐year basis in sites with at least 10 yr of *in situ* fAPAR records (*n* = 3) (Fig. S5) and did not observe any signal of acclimation. While only three sites may not provide sufficient evidence, this observation is consistent with what was reported by Bernacchi *et al*. (2003). It would imply that the acclimation of *V*_cmax_ to temperature, which is a well‐documented phenomenon (Medlyn *et al*., 2002; Kattge & Knorr, 2007), does not apply to $\phi_{0}$. However, some studies have reported acclimation to temperature of the initial slope of the light–response curve, such as Dwyer *et al*. (2007) and Herrmann *et al*. (2020). To our knowledge, only Tenhunen *et al*. (1976a) fitted the same equation as in this study and found variation of $\phi_{0}$ with temperature – but the model presented there assumed constant $\phi_{0}$ for simplicity.

**Fig. 9:**
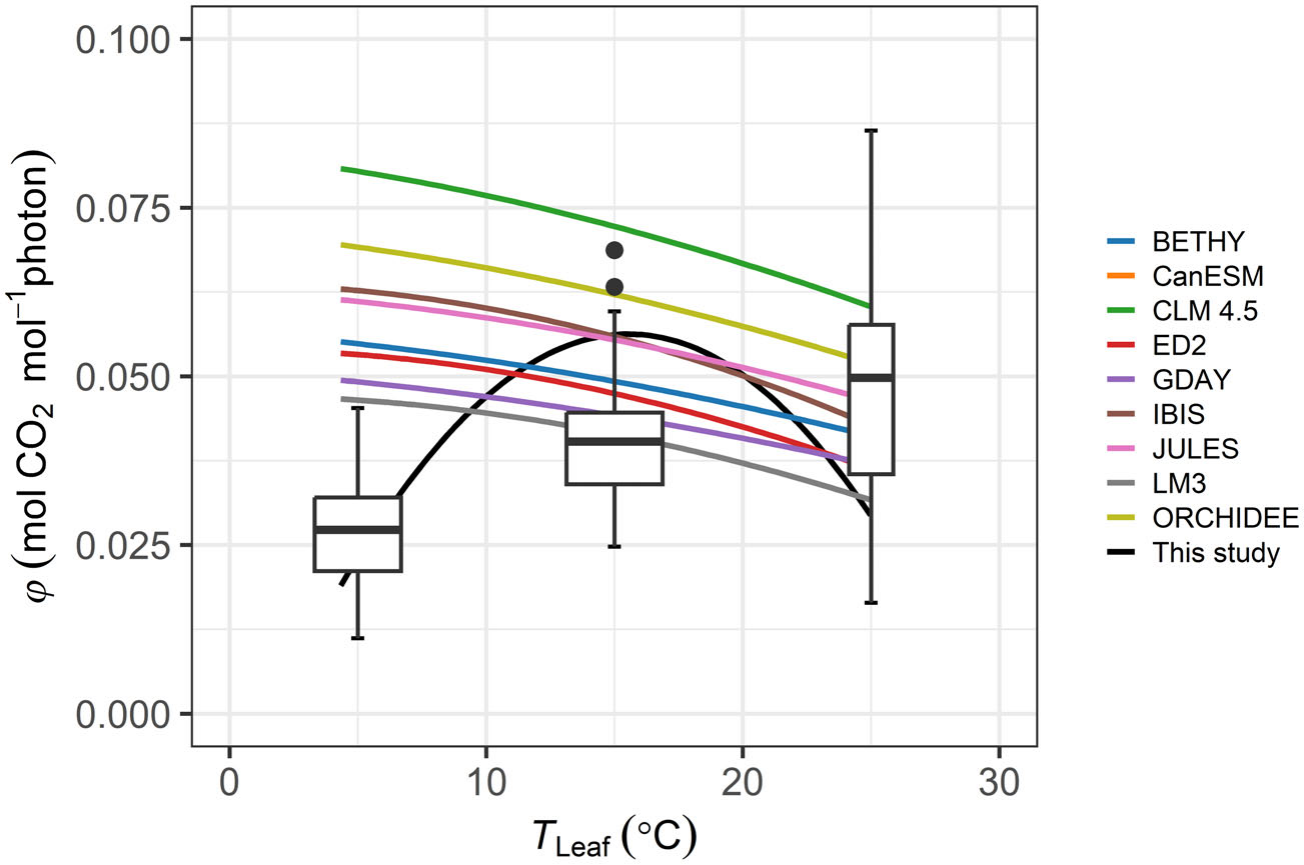
Realised quantum efficiency of primary production measured and predicted by different models for Arctic plants, after Rogers *et al*. (2019); boxes show the interquartile range with the median indicated by the thick solid line. Whiskers extend to the most extreme values within 1.5× the interquartile range, and outliers are shown as black circles. Box widths are proportional to sample size. The thinner black line shows the results of our model. Results of other terrestrial biosphere models (TBMs) (coloured lines) are based on parameters and settings from Rogers *et al*. (2019).

Comparing our predictions with independent observations by Rogers *et al*. (2019) (Fig. 9) shows that considering the temperature dependency of $\phi_{0}$ eliminates a general problem shared by TBMs, of overpredicting $\phi_{0}$ at low temperatures. Our model also predicts a sharper decline in $\phi_{0}$ at temperatures higher than *T*_opt_. All else equal, this translates into a sharper decrease in assimilation at high temperatures.

**Fig. 10:**
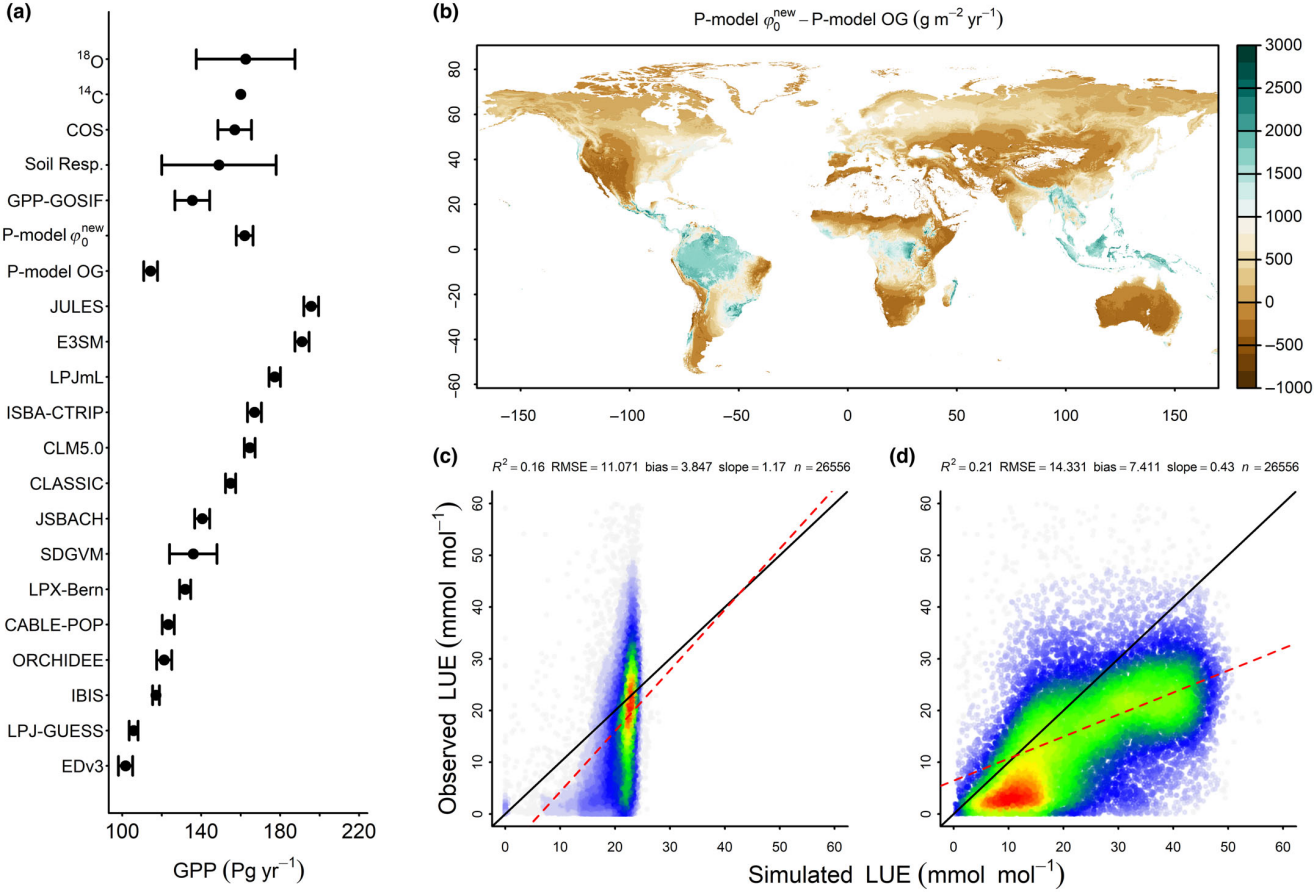
$\phi_{0}$ implementation in the P model. (a) Global mean annual GPP from different sources, including the TRENDY models (Sitch *et al*., 2024) and this study. (b) Spatial distribution of the difference between global mean annual GPP, averaged over the years 2003 to 2020, from the new and the original P model ($\hat{\phi_{0}}=0.087$ and *f* (T) from Bernacchi *et al*., 2003). (c) Correlation of observed and simulated light‐use efficiency (LUE) monthly values using all the flux‐tower data pooled, original P‐model $\phi_{0}$. (d) Correlation of observed and simulated LUE monthly values using all the flux tower data pooled and the new P model $\phi_{0}$. Bars represent standard deviation.

When this new formulation, with global coefficients (Eqs (Eqn 6), (Eqn 7), (Eqn 8), (Eqn 9)), was tested on the P‐model without any soil moisture limitations, we simulated an increase in global total annual GPP from 114.3 ± 3.49 to 161.0 ± 4.38 PgC yr^−1^, with respect to the original. The new value is closer to values inferred from ^14^C (*c*. 160 PgC yr^−1^), ^18^O (150–175 PgC yr^−1^) and carbonyl sulfide uptake, COS (157 ± 8.5 PgC yr^−1^), but far from values derived from inverted soil respiration (149 ± 29 PgC yr^−1^) and inferred from SIF (135.5 ± 8.8 PgC yr^−1^) (Li & Xiao, 2019; Graven *et al*., 2024; Lai *et al*., 2024; Fig. 10a). This change, however, was unevenly distributed; the increase was apparent in the tropical rainforests where the annual GPP increased by 1–3 kgC m^−2^ yr^−1^, while in temperate and boreal forests the increment was moderated to *c*. 0.5 kgC m^−2^ yr^−1^. In arid regions, by contrast, there was a moderate decrease in modelled GPP (Fig. 10b).

When we compared the monthly LUE from the P‐model with the original vs the new $\phi_{0}$ formulation, we saw an improvement in the *R* ^2^ from 0.16 to 0.21 (Fig. 10d). However, the slope and the bias were degraded compared to the original. A possible reason is the fAPAR source. For example, (Stocker *et al*., 2020) found a significant change in the magnitude of GPP when switching from MODIS fAPAR to fAPAR 3 g. In our results, the underestimation occurs for high LUE values, which often represent tropical rainforests – where the error in remotely sensed fAPAR due to constant cloudiness is well‐documented (Zhang‐Zheng *et al*., 2024). It is worth noting that in Stocker *et al*. (2020), the same evaluation was done, but only roughly one‐third of the data was available at that time; their *R* ^2^ was 0.31. In the same way, in our evaluation, the original formulation was not able to surpass *c*. 25 mmol mol^−1^ of monthly LUE (Fig. 10c).

Overall, we have provided global evidence that $\phi_{0}$ is not a fixed parameter (or fixed per plant functional type) as has been widely assumed in TBMs. Instead, it has a bell‐shaped response to temperature, whose parameters are determined by bioclimatic adaptation. Incorporating these responses will be key to reliably assessing the response of the terrestrial biosphere to continuing climate change.

## Competing interests

None declared.

## Author contributions

DS contributed to conceptualisation, coding and first draft writing. VF contributed to scientific input, code supervision, CM contributed to scientific input, ICP contributed to scientific input, supervision. All the authors reviewed and edited the final manuscript.

## Disclaimer

The New Phytologist Foundation remains neutral with regard to jurisdictional claims in maps and in any institutional affiliations.

## Supporting information


**Fig. S1** Apparent and intrinsic quantum yield at leaf and canopy scale leaf‐scale.
**Fig. S2** Correlation of estimated $\phi_{0}$ with NEE and estimated with GPP using the FLUXNET dataset.
**Fig. S3** $\hat{\phi_{0}}$ depicted in climate spaces.
**Fig. S4** Other patterns in the PA parameters.
**Fig. S5** Temporal dynamics of the parameters shaping $\phi_{0}$ (*T*) at selected sites.
**Notes S1** Classic scaling‐up algorithm used by ORCHIDEE.
Please note: Wiley is not responsible for the content or functionality of any Supporting Information supplied by the authors. Any queries (other than missing material) should be directed to the *New Phytologist* Central Office.

## Data Availability

The Eddy covariance data used in this study is openly available from https://ameriflux.lbl.gov/ for the sites with *in situ* fAPAR and FluxDataKit doi: 10.5281/zenodo.12818273 for the sites with matching remote‐sensing fAPAR. The code is available at https://github.com/dsval/Environmental‐influences‐on‐the‐maximum‐quantum‐yield‐of‐terrestrial‐primary‐production.
