# Supplementary material for: Environmental influences on the maximum quantum yield of terrestrial primary production
Source: New Phytol. 2026 May 27;251(4):1709–22. doi: 10.1111/nph.71303 (PMC13373845; doi:10.1111/nph.71303)
Supplement: Supplementary file 1 — Fig. S1 Apparent and intrinsic quantum yield at leaf and canopy scale leaf‐scale. Fig. S2 Correlation of estimated ϕ0 with NEE and estimated with GPP using the FLUXNET dataset. Fig. S3 ϕ0^ depicted in climate spaces. Fig. S4 Other patterns in the PA parameters. Fig. S5 Temporal dynamics of the parameters shaping ϕ0 (T) at selected sites. Notes S1 Classic scaling‐up algorithm used by ORCHIDEE. Please note: Wiley is not responsible for the content or functionality of any Supporting Information supplied by the authors. Any queries (other than missing material) should be directed to the New Phytologist Central Office. [file NPH-251-1709-s001.docx]

## *New Phytologist* Supporting Information

Article title: Environmental influences on the maximum quantum yield of terrestrial primary production

Authors: David Sandoval, Victor Flo, Catherine Morfopoulos and Iain Colin Prentice

Article acceptance date: 06 May 2026

The following Supporting Information is available for this article:

**Notes S1** Classic scaling-up algorithm used by ORCHIDEE.

**Fig. S1** Apparent and intrinsic quantum yield at leaf and canopy scale leaf‑scale.

**Fig. S2** Correlation of estimated $\varphi_{0}$ with NEE and estimated with GPP using the FLUXNET dataset.

**Fig. S3** $\hat{\varphi_{0}}$ depicted in climate spaces. (a) $\hat{\varphi_{0}}$ in the space aridity-growth temperature. (b) $\hat{\varphi_{0}}$ in the Budyko space.

**Fig. S4** Other patterns in the PA parameters. **a** partial residuals plot of $\hat{\varphi_{0}}$ and AI. **b** partial residuals plot of $\hat{\varphi_{0}}$ and mGDD_0._

**Fig. S5** Temporal dynamics of the parameters shaping ϕ_0_ (T) at selected sites.

**Notes S1** Classic scaling-up algorithm used by ORCHIDEE (Naudts et al., 2015)

Below, we provide an analytical description of the scaling-up algorithm used by ORCHIDEE (Naudts et al., 2015). Followed by simulations carried out with a 20-layer canopy (LAI = 5), a Beer–Lambert extinction coefficient of 0.5, and an exponential decline of *J*_max_ with depth (assuming an extinction coefficient of 0.15). Note that the vertical gradient of canopy temperature is disregarded here (a reasonable approximation for low PAR, with expected variations ≤ 1 K: Song et al., 2017) so we do not consider any temperature-related variations of *J*_max_, *V*_cmax_ and *m*_j_ through the canopy.

We define terms as follows:

- - $\varphi_{0,leaf}$ is the leaf‑scale intrinsic quantum yield;
  - $\varphi_{0,can}$: is the canopy‑scale intrinsic quantum yield.

Then we have:

$J_{leaf}\approx\varphi_{0,leaf}.PPFD,when PPFD\to0$

$$J_{can}\approx\varphi_{0,can}.APAR,when APAR\to0$$

where *J* is the electron transport rate, PPFD is the absorbed photosynthetic photon flux density (by a leaf), and APAR is the absorbed photosynthetically active radiation (by the canopy). Under low light and assuming the Beer-Lambert extinction law,

$A_{leaf}\left( z \right)=\frac{\varphi_{0,leaf}}{4}m_{j}PPFD\left( z \right),with PPFD\left( z \right)={PPFD}_{o}e^{-kz}\wedge z\in\left[ 0,LAI \right]$

$$A_{can}=\int_{0}^{LAI} A_{leaf}dz=\int_{0}^{LAI} \frac{\varphi_{0,leaf}}{4}m_{j}PPFD\left( z \right)dz$$

where *z* is depth in the canopy, and *m*_j_ is the term accounting for photorespiration. Since

$$APAR=\int_{0}^{LAI} PPFD\left( z \right)dz={PPFD}_{o}\frac{1-e^{-k.LAI}}{k}$$

we have

$A_{can}=\int_{0}^{LAI} \frac{\varphi_{0,leaf}}{4}m_{j}PPFD\left( z \right)dz=\frac{\varphi_{0,leaf}}{4}m_{j}APAR$

and by definition:

$$A_{can}\approx\frac{\varphi_{0,can}}{4}m_{j}.APAR,when APAR\to0$$

so comparing coefficients,

$$\frac{\varphi_{0,can}}{4}m_{j}.APAR=\frac{\varphi_{0,leaf}}{4}m_{j}.APAR$$

resulting in $\varphi_{0,leaf}=\varphi_{0,can}$.

Importantly, vertical declines of $J_{max}$ or $V_{cmax}$ affect the curvature and saturation levels of light-response curves but they do not alter the slope of $A_{can}\frac{4}{mj}$, which is set by $\varphi_{0,leaf}$ – as shown in the Figures below.

**Fig. S1** Apparent and intrinsic quantum yields at leaf (a, c) and canopy (b, d) scales, across temperatures (10, 20, 30, 40 °C) versus PPFD (a, b) and PPFD.*m*_j_/4 (c, d). Vertical lines in panels c and d show the value of PPFD.*m*_j_/4 corresponding to a PPFD of 150 µmol m^−2^ s^−1^. Electron transport follows a non‑rectangular hyperbola; temperature responses of *J*_max_ and Γ^•^ are from ORCHIDEE (Naudts et al., 2015). *J*_max_25_ was set at 120 µmol m^−2^ s^−1^.

**Fig. S2** Correlation of estimated $\varphi_{0}$ with NEE and estimated with GPP using the FLUXNET dataset.


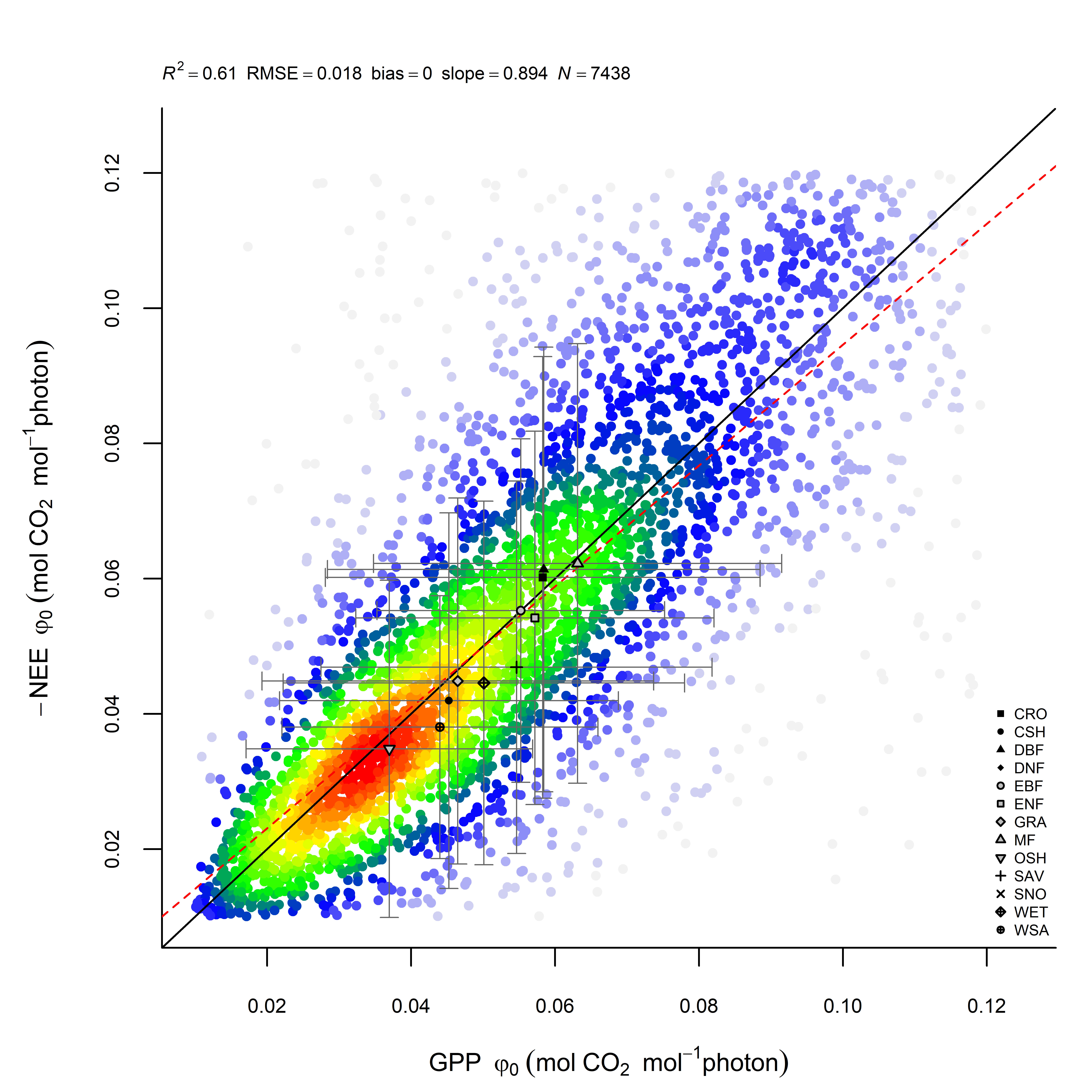


**Fig. S3** $\hat{\varphi_{0}}$ depicted in climate spaces. (a) $\hat{\varphi_{0}}$ in the space aridity-growth temperature. (b) $\hat{\varphi_{0}}$ in the Budyko space


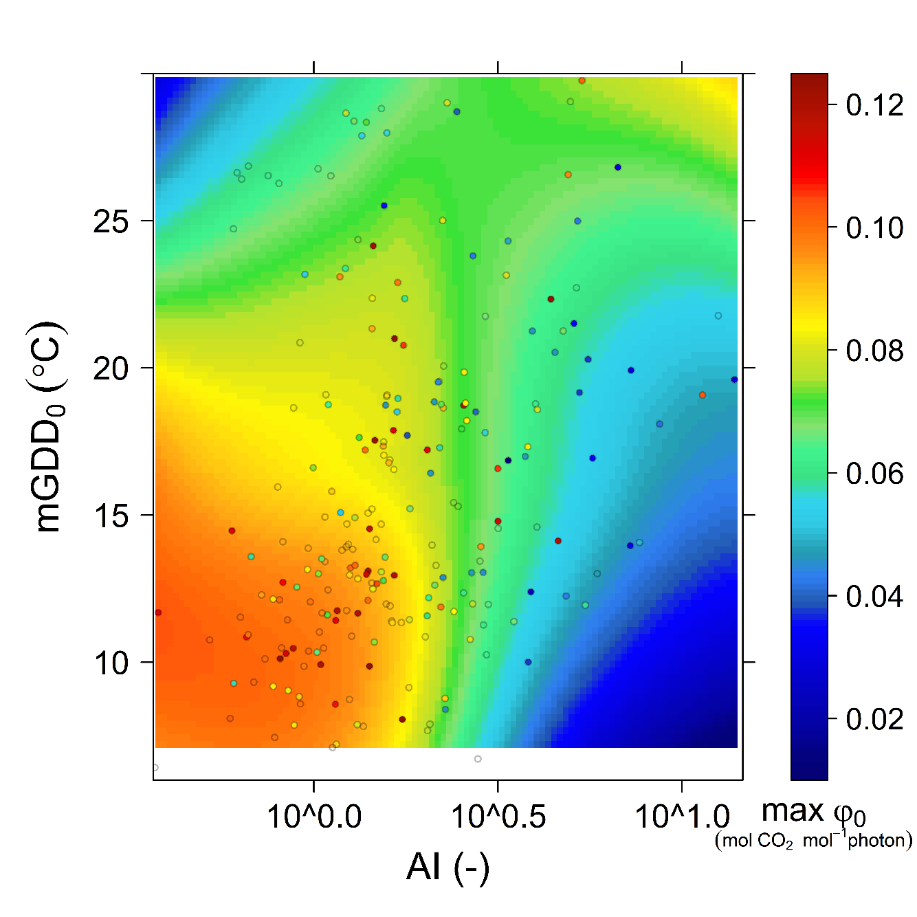

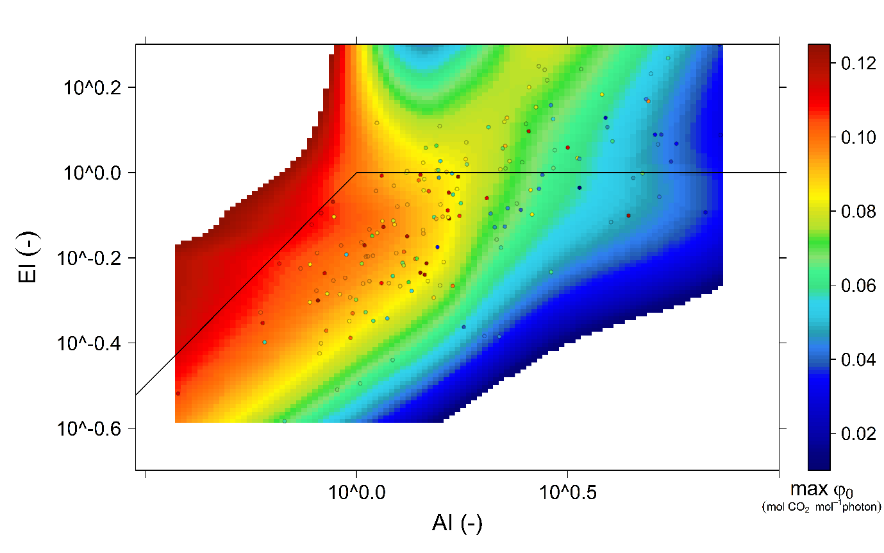


**Fig. S4** Other patterns in the PA parameters. **a** partial residuals plot of $\hat{\varphi_{0}}$ and AI. **b** partial residuals plot of $\hat{\varphi_{0}}$ and mGDD_0_.


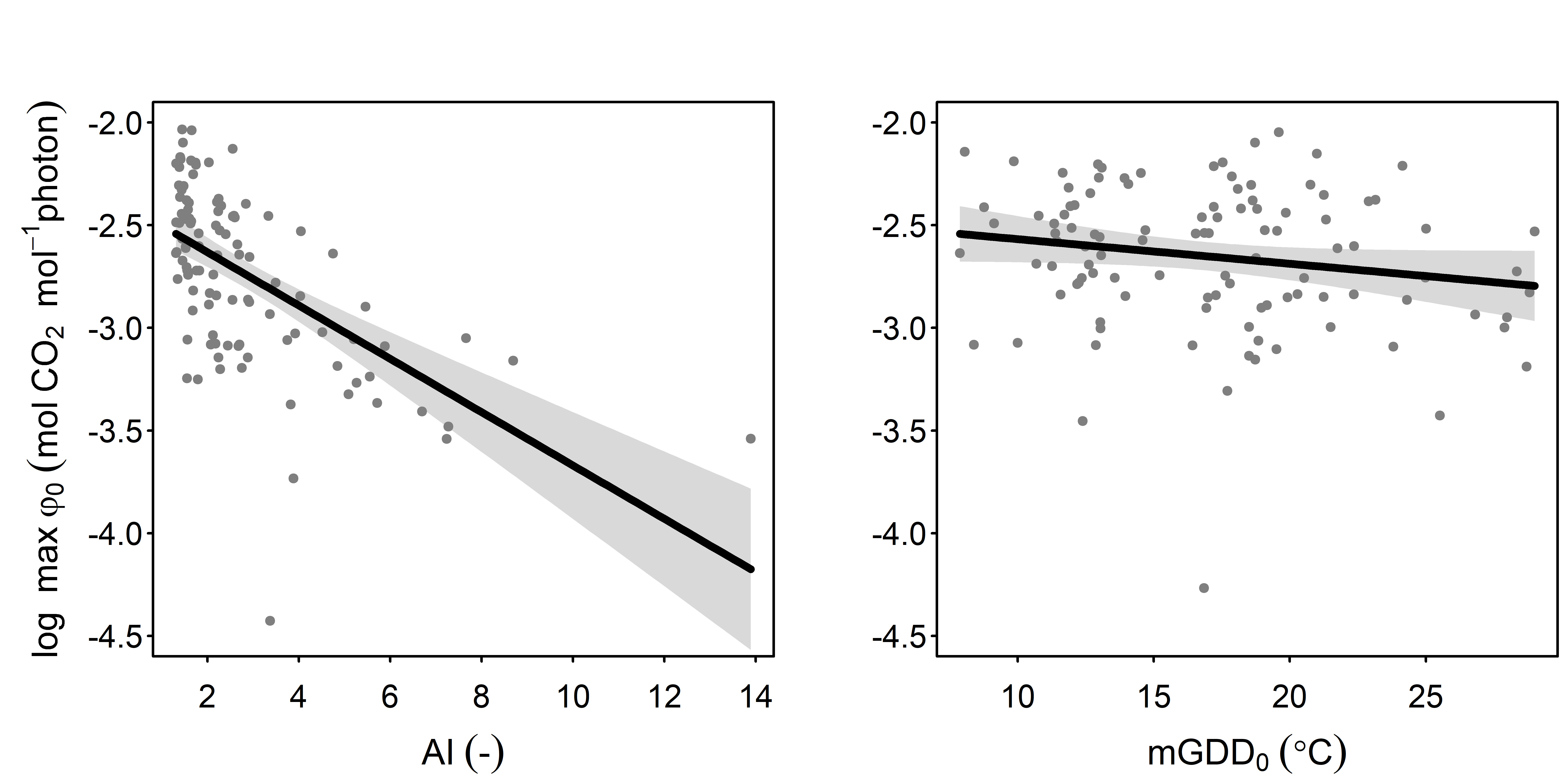

 **Fig. S5** Temporal dynamics of the parameters shaping ϕ_0_ (T) at selected sites

**References:**

Naudts, K., Ryder, J., McGrath, M. J., Otto, J., Chen, Y., Valade, A., Bellasen, V., Berhongaray, G., Bönisch, G., Campioli, M., Ghattas, J., De Groote, T., Haverd, V., Kattge, J., MacBean, N., Maignan, F., Merilä, P., Penuelas, J., Peylin, P., … Luyssaert, S. (2015). A vertically discretised canopy description for ORCHIDEE (SVN r2290) and the modifications to the energy, water and carbon fluxes. Geoscientific Model Development, 8(7), 2035–2065. https://doi.org/10.5194/gmd-8-2035-2015

Lambers H, Chapin FS III, Pons TL (2008) Plant Physiological Ecology. 2nd edn. Springer, New York. ISBN: 978‑0387783406. Chapters: The Plant’s Energy Balance; Scaling‑Up Gas Exchange and Energy Balance from the Leaf to the Canopy Level.

Song Q‑H, Deng Y, Zhang Y‑P, Deng X‑B, Lin Y‑X et al. (2017) Comparison of infrared canopy temperature in a rubber plantation and tropical rain forest. International Journal of Biometeorology 61: 1885–1892. <https://doi.org/10.1007/s00484-017-1375-4>.
